# Mechanisms of Immune Cell Dysregulation in Pancreatic Ductal Adenocarcinoma

**DOI:** 10.3390/biology15181599

**Published:** 2026-09-10

**Authors:** Farah Ahmady-Nield, Rodney B. Luwor, George Kannourakis

**Affiliations:** 1 Fiona Elsey Cancer Research Institute, Ballarat, VIC 3350, Australia; 2 Institute of Innovation, Science and Sustainability, Federation University Australia, Mt Helen, Ballarat, VIC 3350, Australia; 3 Department of Surgery, The University of Melbourne, The Royal Melbourne Hospital, Parkville, VIC 3050, Australia

**Keywords:** pancreatic ductal adenocarcinoma, tumor–immune–stromal microenvironment, immune cells, immune dysregulation

## Abstract

Pancreatic ductal adenocarcinoma (PDAC) is an aggressive form of pancreatic cancer with low five-year overall survival rates of ~10% from diagnosis. The immune ‘cold’ tumor microenvironment (TME) of PDAC is a key contributor to tumor progression, featuring immunosuppression in conjunction with a reactive stroma. The immune composition of the TME is highly tumor-promoting, with the presence of immunosuppressive cells and the absence of effector and cytotoxic immune cells. Further to this, the active stroma region of the tumors contains transformed fibroblasts which communicate with tumor and immune cells to further enhance immunosuppression. Fundamental cellular processes such as T cell exhaustion, metabolic signaling, epigenetic modifications, and a series of genetic mutations can all contribute to PDAC progression and poor patient outcomes. This review describes the key mechanisms behind immune dysregulation in PDAC, highlighting the importance of this area and the essential research that is necessary.

## Simple Summary

Pancreatic cancer is an aggressive disease and a leading cause of cancer-related deaths. Cells are able to undergo changes in the tumor, which is correlated with reduced efficacy of the immune system to target and destroy the tumor. This is a leading factor contributing to the severity of the cancer and requires further study. By understanding these factors, potential new treatment outcomes incorporating the immune system may be discovered. This review summarizes the currently known relationship between pancreatic cancer and the immune system.

## 1. Introduction

Pancreatic cancer is a highly aggressive disease and a leading cause of cancer-related death worldwide. The severity of the disease is contributed to by several factors such as advanced-stage diagnosis in patients, resistance to treatment and the immunosuppressive tumor microenvironment (TME). Thus, survival rates in patients remain troublesome. The worldwide five-year overall survival (OS) rate for pancreatic cancer is only ~10% [1], highlighting the seriousness of the disease.

Pancreatic tumors are divided into two main groups with distinct phenotypes: exocrine tumors and pancreatic neuroendocrine tumors (NETs) [2]. Exocrine tumors comprise the majority of pancreatic cancers, with a 90–95% incidence, compared to NETs, which make up the remaining ~5% [3]. Within these two groups, there are several subtypes, with pancreatic ductal adenocarcinoma (PDAC) comprising the majority (~90%) of primary pancreatic cancer cases and being the most common but also highly aggressive form of pancreatic cancer [4,5].

Treatment for PDAC remains problematic, as surgery is the only curative approach and only 10–15% of PDAC tumor cases can be resected [6,7]. Tumors are characterized by resectability status: resectable, borderline resectable and locally advanced. Depending on which group the tumor falls under dictates what treatment plan is given. The resectable group is given surgery followed by chemotherapy; borderline resectable tumors are given neoadjuvant chemotherapy, restaged, and then given surgery if possible. Locally advanced cases can be managed with a non-surgical approach due to the risk and are given systemic chemotherapy +/− radiation [8]. It is only if there is a response to initial chemotherapy that surgical resection may be considered. For distal metastasis cases, resection is not often an option. In very specific cases of PDAC, there have been resections of tumors from organs such as the liver and aortocaval lymph nodes with links to improved survival [9]; however, it is not standard of care for this cohort of patients and further study is necessary.

Over the last decade, immunotherapy has been successful in revolutionizing cancer treatment [10]. Although mono-immunotherapy and combination immunotherapy have shown modest results in pancreatic cancer trials, including those who are microsatellite instability-high or mismatch repair-deficient, it remains largely unsuccessful [11,12,13,14,15]. This is a result of several factors such as the immunosuppressive TME, large stromal component and low expression of antigen [13,16].

For several years, numerous clinical studies have been conducted to determine better, more refined ways to treat PDAC, some of which show promise, as reviewed by Hayat et al. [12]. One recently refined approach is the utilization of specific KRAS inhibitors, which has revealed real potential for the treatment of PDAC. Kirsten rat sarcoma viral oncogene homolog (*KRAS*) mutations are present at extremely high rates (~95%) in PDAC tumors and have been studied as a potential target for decades due to their ability to modulate the TME of PDAC [17,18]. Recently, researchers and clinicians have found a way to effectively target this pathway, and results have shown a two-fold increase in median OS as compared to chemotherapy-treated patients [19]. This is yet to be incorporated into the standard of care, and further study is required. One of the key reasons why treatment can be troublesome, and patients can become resistant in aggressive cancers including PDAC, is the dysregulation of the immune system and the lack of thorough understanding of the mechanisms behind this dysregulation.

**Figure 1:**
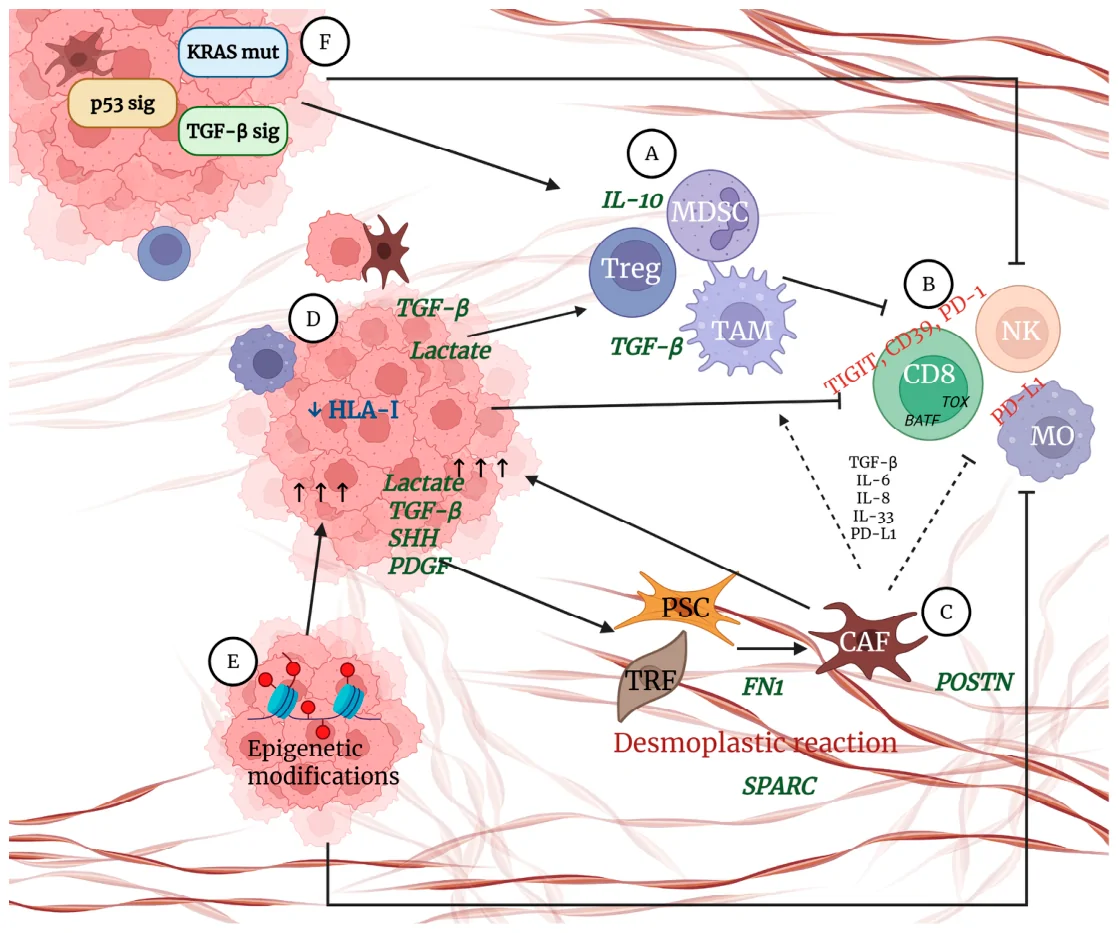
The multi-networking system resulting in immune dysregulation and immunosuppression in PDAC. The PDAC TME is comprised of an abundance of immunosuppressive immune cell subsets such as T regulatory cells (Tregs), tumor-associated macrophages (TAMs) and myeloid-derived suppressor cells (MDSCs), which can produce suppressive cytokines/factors such as TGF-β and IL-10 (A). Anti-tumor immune cell subsets such as CD8+ T cells, NK cells and macrophages (B) are found in lower numbers, a result of several different processes in the PDAC TME. (C) Desmoplastic reaction, which is a process resulting in stromal ‘stiffness’, is formed by cells such as pancreatic stellate cells (PSCs) and tissue-resident fibroblasts (TRFs) transforming into different types of CAFs, largely immunosuppressive in nature, and located at various distances from the tumor site. Tumor PDAC cells communicate with CAFs via the release of factors such as lactate, TGF-β, sonic hedgehog (SHH) and platelet-derived growth factor (PDGF) (D) to promote the formation of the cell type, and CAFs can communicate in return to promote the growth of cancer cells (↑↑↑) via extracellular matrix (ECM) such as fibronectin 1 (FN1), periostin (POSTN) and SPARC. CAFs also secrete a range of different factors such as TGF-β and PD-L1, which can promote suppressive immune cells (A) and inhibit the anti-tumor immune response (B). PDAC cells and suppressive immune subsets such as TAMs produce factors (i.e., TGF-β), which upregulate the expression of checkpoint molecules (e.g., TIGIT, CD39, PD-1 and PD-L1) along with modifications in key transcriptional factors such as basic leucine zipper ATF-like transcription factor (BATF) and thymocyte-associated high mobility group box (TOX), contributing to an exhausted phenotype of anti-tumor immune cells such as CD8+ T cells and macrophages. Epigenetic modifications (E) such as DNA methylation, histone modification, chromatin remodeling and modifications to human leukocyte antigen class I (HLA-I) expression in PDAC cells are all ways the tumor can promote its growth and escape immune evasion via blocking the function of anti-tumor immunity (B) and, in some cases, interacting with CAFs. Key driver mutations and signaling pathways in PDAC, such as KRAS mutation (KRAS mut), TGF-β signaling and p53 signaling (F), enhance the survival of PDAC cells, promote the function of CAFs (C) and suppressive immune cell subsets (A), and inhibit the function of anti-tumor immunity (B). These processes in PDAC work hand in hand to enable immune evasion and promote immunosuppression. Image created in BioRender.com.

Immune dysregulation in PDAC patients is a result of a multi-network system with various immune evasion strategies and angles. These include an abundance of immunosuppressive cellular subsets, absence of anti-tumor immune cells at the tumor site, stromal remodeling resulting in desmoplastic reactions and progressive T cell dysfunction. Other factors such as metabolic competition, epigenetic modifications such as DNA methylation, histone modification and chromatin remodeling, and the initiation of key driver mutations and signaling pathways such as KRAS, transforming growth factor-beta (TGF-β) and p53 also play a key role [20,21,22,23,24]. These biological processes work hand in hand to create an immune ‘cold’ microenvironment in PDAC, reducing the function of anti-tumor immune subsets and recruiting more suppressive cells such as tumor-associated macrophages (TAMs) and cancer-associated fibroblasts (CAFs) (Figure 1). This review will individually break down the key mechanisms involved in immune cell dysregulation in PDAC.

## 2. Immune Cells in the TME

**Figure 2:**
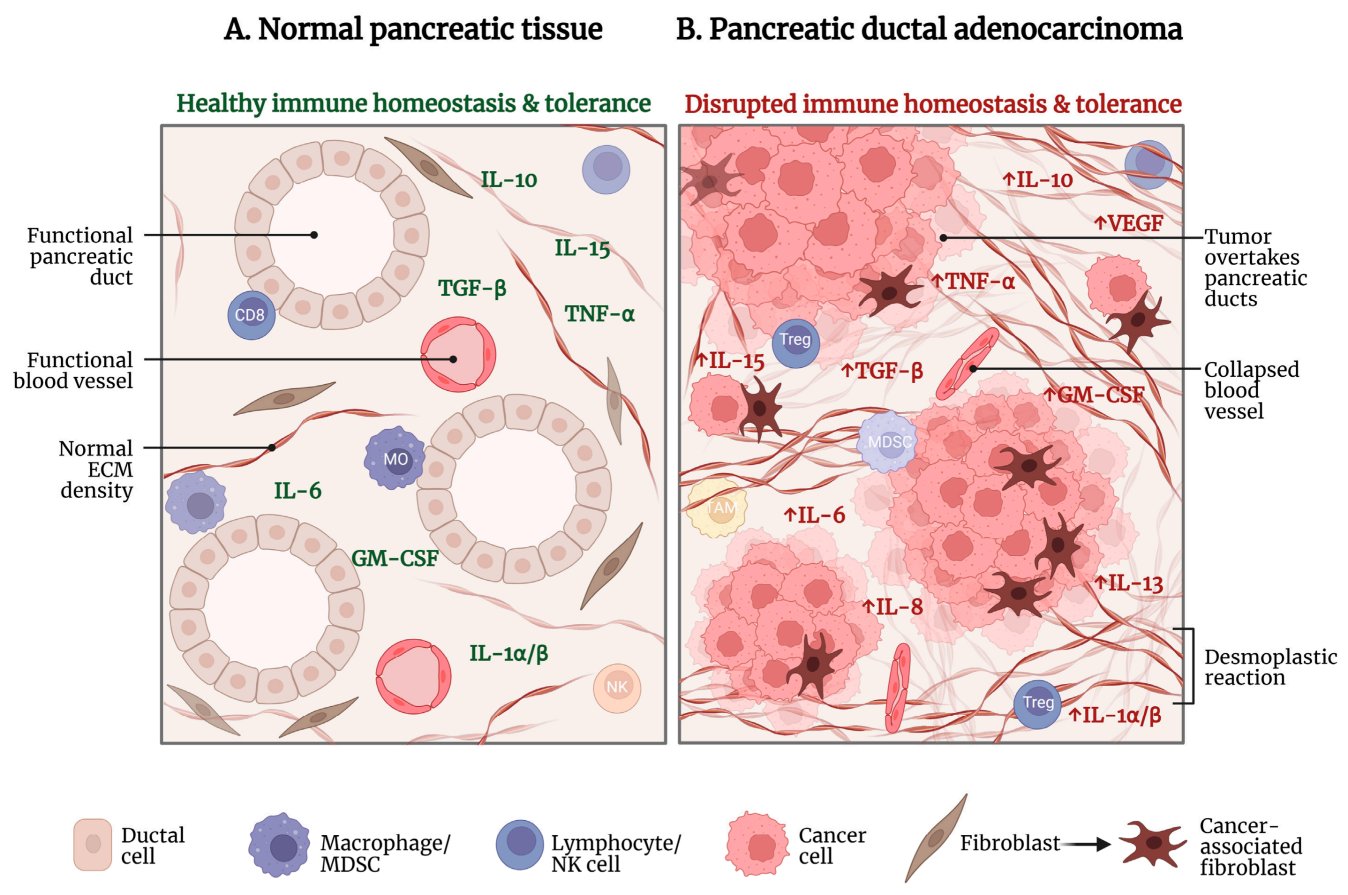
A comparison of the microenvironment milieu of normal pancreatic tissue and PDAC. (**A**) Representation of normal pancreatic tissue with healthy immune homeostasis and tolerance. The cellular composition of normal pancreatic tissue consists of functional ductal cells, immune cells such as lymphocytes (CD8+ T cells) and macrophages (MO) and stromal cells such as fibroblasts. Extracellular matrix (ECM) density, functional blood vessels and pancreatic ducts are maintained with cellular interactions and normal concentrations of key secretory factors such as IL-6, TNF-α, and TGF-β, to name a few. (**B**) Representation of PDAC with disrupted immune homeostasis and tolerance. PDAC is largely comprised of tumor cells that overtake pancreatic ducts, an abundance of suppressive immune cell subsets such as Tregs, MDSCs and TAMs, and a stromal component, which is largely comprised of cancer-associated fibroblasts (CAFs). These cell types and their interconnecting relationships result in a highly immunosuppressive microenvironment milieu, with an increase in TGF-β, GM-CSF, IL-6 and IL-10, to name a few. ↑ represents elevation and ↓ represents reduction. Image created in BioRender.com.

Like other aggressive cancers, the TME of PDAC is highly immunosuppressive and pancreatic tumors are labeled ‘cold’. Although PDACs have a large stromal component and are ‘cold’, the TME still contains a range of different innate and adaptive immune subsets; thus, the tumor is quite heterogeneous [25]. The TME contains some tumor-infiltrating T cells (TILs) such as CD4+ and CD8+ T cells [26], fewer cytotoxic cells such as natural killer (NK) cells [27], but also several suppressive immune cell subsets including TAMs, myeloid-derived suppressor cells (MDSCs), M2 macrophages, N2 neutrophils, T regulatory cells (Tregs), B regulatory cells (Bregs), CAFs and suppressive NK cells [28,29,30]. These suppressive cells produce elevated levels of factors such as IL-10, TGF-β, and granulocyte-macrophage colony-stimulating factor (GM-CSF) [31]. Factors contributing to the immunosuppressive signature can be produced by both PDAC cells, causing an upregulation of these immunosuppressive cell subsets [32], and immune cells (refer to Figure 1). In PDAC cases where TILs are found in higher abundance, they are linked to favorable patient outcomes [33,34,35] and emphasize the need to skew the largely immunosuppressive PDAC TME to a more anti-tumor environment. Figure 2 depicts the difference in the immune microenvironment milieu of normal pancreatic tissue compared with PDAC.

Several recent studies have aimed to dissect the role and spatial location of immune cells, utilizing sequencing and spatial omics techniques [36,37,38,39,40,41]. Sivakumar et al. have recently profiled PDAC tumors at a gene level for immune infiltration. Single-cell RNA-seq, ADT-seq, B cell receptor and T cell receptor seq were initially performed on CD45+ enriched immune cells from treatment-naïve human PDAC tumors and their matched PBMCs [40]. This dataset was labeled PancrImmune and analyzed alongside the published human datasets from Peng [25] and Steele [42]. The overarching findings of this analysis were that the TME had a high level of complexity and functional immune cell infiltration, both activated and regulatory in nature [40]. Taking into consideration that patients with higher intra-tumoral T cells have better survival outcomes, this study aimed to determine the mediating factors for these patient outcomes. To do so, the patients were divided into adaptive-enriched (AE) based on a higher percentage of B and T cell infiltration and myeloid-enriched (ME) based on high myeloid and lower percentage of B and T cell infiltration. Higher plasma cell abundance was present in the ME group as compared to AE, and there were no differences in the proportion of non-immune cell subsets between AE and ME groups, suggesting that the mechanisms are immune cell-driven.

It is a known phenomenon that in the TME, the location of immune cells in proximity to the tumor cells is an indicator of patient outcome. When analyzing human PDAC tumors, Ene-Obong et al. found that key immune cell subsets such as NK cells, B cells, CD8+ T cells, and FOXP3+ Tregs were largely present in the pan-stromal compartment and were not present near the tumor cells, as shown by the inability of immune cells to penetrate the juxtatumoral stromal region [41]. The localization of these immune cell subsets in the pan-stromal compartment could be a result of the desmoplastic reaction, a concept discussed in more detail in Section 3, that occurs in the stroma, resulting in these cells becoming ‘stuck’. However, macrophages, which can possess pro-tumor properties, were present in the juxtatumoral region, and studies have shown that fibroblast activation protein (FAP) produced by CAFs can cleave collagen, resulting in macrophage binding, promoting a pro-tumor response [43]. When linking these findings to survival, further proximity studies have shown that patients benefit when TILs are closer to the tumor cells. Patients with lower immunogenicity were found to have fewer TILs near the tumor cells and greater high-grade tumor budding, as compared to the higher immunogenicity group [37,38]. This highlights the importance of tumor–immune cell interactions in the containment of PDAC and will be discussed further in Section 3. By determining mechanisms to transform the ‘cold’ immunosuppressive PDAC TME to a ‘hot’ anti-tumor immune-rich environment, it may lead to improved patient outcomes for what is a very aggressive disease.

## 3. Stromal to Tumor–Immune Crosstalk in the TME

As the TME is largely composed of immune, stromal and tumor components, the crosstalk between the cell types is important in understanding the progression of PDAC. More recently, the importance of a reactive stromal region of PDACs has been established, with stromal cells interacting with tumor and immune cells to shape the TME and resulting in PDAC progression. Cell-to-cell contact and secretory-based communications are pivotal ways intercommunication can take place.

Gene analysis studies have discovered various signaling pathways in key interactions between tumor cells and the TME in PDAC. Studies have molecularly subtyped human PDAC tumors [44,45,46] into classical, quasi-mesenchymal and exocrine-like [44], and others into squamous, pancreatic progenitor, aberrantly differentiated endocrine exocrine, and immunogenic [45]. However, a major limitation in these studies is the contamination of PDAC tissue with surrounding pancreatic tissue; thus, the analysis is not entirely representative of a PDAC-pure population. To address this issue, a human study by Moffitt et al. categorized and dissected the samples into normal, tumor and microenvironment to obtain true molecular subtypes [47]. From this analysis, two PDAC subtypes were identified: basal-like and classical, and profiles specific to activated stroma based on expression of extracellular matrix (ECM) proteins such as periostin (POSTN), secreted protein acidic and rich in cysteine (SPARC) and fibronectin 1 (FN1) [47]. These profiles were further linked to PDAC progression, thus highlighting the importance of the stromal components of cancer. Five years later, a study by Hiroshima et al. found that this stromal signature was apparent and the specific proteins were differentially expressed in the stromal component of PDAC tumors. This study micro dissected pancreatic cancer, adenoma, normal ducts, acinar cells and islets to remove contamination [48]. Differentially expressed genes (DEGs) from stromal tissue of PDACs and DEGs from PDAC cells were compared. From this analysis, nine genes and eight key cancer–stromal-interaction targets and their relevant signaling pathways in the interaction between PDAC and the surrounding stromal cells were identified [48]. These data were obtained from an in-house ligand–receptor interaction database where interactions between cancer ligands and stromal receptors and stromal ligands and cancer receptors were evaluated [48]. The only interactions determined were in the stromal ligand-to-cancer receptor direction, and ECM genes, thrombospondin 1 (*THBS1*), tenascin (*TNC*), *FN1*, and various subunits of integrin (ITG) (*ITGB1*, *ITGB3*, *ITGB6*, *ITGB7*, *ITGA3* and *ITGA5*) were identified [48]. Survival experimental studies revealed that co-expression of the genes linked to the eight key interactions significantly reduced OS in PDAC patients for *FN1-ITGA3* and *FN1-ITGA5* interactions, and IHC studies confirmed that FN1 was a stromal ligand and the *ITGA3* receptor was largely expressed by PDAC tumor cells [48]. *ITGA3* is a previously identified prognostic and diagnostic biomarker for PDAC [49]; thus, the study by Hiroshima et al. found a potential mechanism behind the elevation of this marker.

When dissecting stromal cellular makeup in PDAC, CAFs, endothelial cells and infiltrating immune cells are present, with CAFs being the most abundant and key player subset originating from pancreatic stellate cells (PSCs), tissue-resident fibroblasts and tumor-infiltrating mesenchymal stem cells [50,51]. CAFs are transformed and activated fibroblasts in the stromal component of tumors with different precursor cell origins and have long been associated with cancer progression via ECM remodeling and modulating the TME [52,53]; the same is also true for PDAC [54]. Moreover, CAFs are signaled by tumor cells to become key contributors to the PDAC desmoplastic reaction, which causes a thick layer of fibrous connective tissue via the release of extra collagen and proteins surrounding the PDAC cells [20]. Moreover, CAFs can inhibit the function of cytotoxic T cells, a key mechanism of reducing anti-tumor immunity [55]. Highlighting their importance, CAFs can contribute to treatment failure by promoting chemotherapy resistance, i.e., gemcitabine resistance in pancreatic cancer cells, through the secretion of cytokines such as IL-6 [56].

It is established that CAFs play an important role in manipulating tumor immunity. There are several subtypes of CAFs, each of which plays a unique role in the context of cancer, with varied phenotypic and functional status, whether it be pro- or anti-tumor. The plasticity of CAFs is based on CAF precursors, cancer type and tumor heterogeneity [57]. Some of the key CAFs identified include myofibroblastic (myCAFs), inflammatory (iCAFs), and antigen-presenting immunomodulatory (apCAFs), emphasizing the heterogeneity of the cell population.

In 2017, Ohlund et al. found that myCAFs and iCAFs are the two key CAF subsets present in PDAC, with further human and mouse studies confirming this [58]. Spatial studies located myCAFs in the periglandular region, where direct interactions with tumor cells were suggested to be important for their formation. They were defined by high alpha-smooth muscle actin (α-SMA) and low IL-6 expression and a lack of inflammatory cytokine expression [58]. iCAFs are induced by secretory paracrine factors of tumor cells such as IL-1α and TNF-α [58,59] and have been located distant from the tumor cells and myCAFs and express lower levels of α-SMA and produce higher levels of inflammatory cytokines such as IL-6 and IL-11 [58]. The plasticity of CAFs is highlighted by myCAFs ability to produce both pro- and anti-tumor responses, depending on factors such as tumor stage and the TME, as well as the ability of iCAFs to transform into myCAFs, based on specific signals such as TGF-β, present in the TME [58,60]. Since the identification of these two CAF populations, Elyada et al. found that apCAFs are also present in PDAC, expressing MHC-II molecules and CD74 [61] and are associated with high levels of Tregs and reduced anti-tumor immunity in PDAC. In culture, apCAFs can also transform into myCAFs, highlighting this specific CAF population as a key player and source of origin [61].

In the PDAC TME, CAFs can interact with several cell types via cell adhesion, antigen presentation, metabolic pathways and the release of cytokines and chemokines, thus modulating the TME [62,63] (refer to Figure 1). Additionally, stromal cells can share activation status of the same signaling pathways as tumor cells, which can lead to cancer progression, emphasizing their cohesion with tumor cells. An example of this is the activation of STAT3 signaling in both epithelial tumor cells [64] and in CAFs by tumor cells in PDAC [65]. This highlights the relationship between tumor and stromal components of PDAC.

When investigating mechanisms behind stromal-tumor crosstalk, Begum et al. demonstrated that CAFs and cancer cells can communicate via cell-to-cell contact [66]. Clonogenic growth was enhanced when human CAFs were co-cultured with PDAC cells in vitro and remained unchanged when PDAC cells were co-cultured with CAF-conditioned media [66]. In addition to clonogenic growth, PDAC cells became more migratory when exposed to CAFs, suggesting an epithelial-to-mesenchymal (EMT) mechanism contributing to metastatic PDAC, both via cell-to-cell contact and via CAF secretory factors such as MMP11 [66,67,68]. PDAC cells can also influence fibroblasts by producing factors such as TGF-β, sonic hedgehog (SHH) and platelet-derived growth factor (PDGF), which can promote ECM accumulation and the growth of CAFs, thus contributing to greater tumor progression [69,70,71,72].

In PDAC, CAFs not only interact with tumor cells, but also with various immune cell subsets in the TME, modulating their responses. Zhang et al. have thoroughly outlined the immunomodulatory effects of CAFs in the PDAC TME [63]. CAFs are able to produce a range of additional cytokines and factors such as TGF-β, CXCL12, IL-8, IL-33, M-CSF, PD-L1 and AGR2, which can modulate immune cell subsets such as CD8+ T cells, Tregs, NK cells, MDSCs, macrophages and neutrophils [63,73], shaping them to enhance immunosuppression in the TME. CAFs in vitro are also able to release exosomes such as miR-181b-5p, which inhibit STING signaling, a key type I interferon response pathway, in pancreatic cells, thus leading to immune evasion and immune cell inhibition by tumor cells [74]. STING itself can be expressed in tumor cells, immune cells and CAFs. Mouse model studies have shown that STING agonists used in tumors with high stromal STING expression compared to low could be more effective [75]. In KPC (PDAC developing) mice treated with STING agonist DMXAA, STING was phosphorylated in both tumor and stromal cells, and this was linked to increased levels of IFN-β, TNF-α, CXCL1, CXCL10 and IL-6, indicative of an active immune response [75]. IHC studies revealed significantly more CD8a immune cells compared to the control. Further to functional outputs, the mRNA levels of granzyme B and perforin were significantly higher as determined by qPCR [75]. Although these studies mention the potential relevance of these findings in human PDAC, experiments utilizing human PDAC samples are necessary to determine if this is the case.

Additional ways CAFs can be associated with immunosuppression are via their ability to enclose CD8+ T cells at the periphery of tumors to avoid contact with tumor cells [41] and upregulate immune checkpoint proteins on their surface or on extracellular vesicles, causing T cell inhibition [63]. Although CAFs largely play a role in immunosuppression, mouse studies have shown that the depletion of α-SMA + myCAFs disrupts the immune balance and keeps cells like Tregs upregulated in PDAC, with PDAC patients with lower myCAFs correlating with reduced survival [76]. Moreover, depletion of myCAFs transformed the ECM composition, resulting in reduced type I collagen [76]. Further studies are necessary to determine whether other CAF populations compensate for one another and to determine the potential and specific mechanisms behind skewing CAFS to elude an anti-tumor response and thus ways they could be incorporated for PDAC patients.

## 4. Immune Cell Dysregulation in the TME

Immune cell dysregulation can encompass various additional processes, including immune cell exhaustion, metabolic alterations and epigenetic modifications. These processes are used as pro-tumor tactics in cancer, resulting in immune evasion by the tumor, and can act as compensatory mechanisms to promote immunosuppression. These compensatory actions may explain the reduced efficacy of immunotherapy in PDAC.

### 4.1. T Cell Exhaustion

Exhaustion of immune cells has been well described in T cells and defined as a state of differentiation triggered by chronic antigen exposure to the T cell receptor (TCR), a phenomenon that can naturally occur with age, but can also occur through exposure to chronic infections and cancer [77]. Upon normal activation of T cells, activation-induced markers (AIMs) such as CD69, CD25, CD28 and CD154 are upregulated and are indicative of a functional T cell [78,79,80,81,82]. In addition to these receptors, the expression of co-inhibitory checkpoint proteins such as PD-1/PD-L1, CTLA-4, TIM-3 and LAG3 is also present to mitigate overactivation and chronic inflammation, conserve T cell tolerance, and can work as an ‘off’ switch [83,84]. T cell exhaustion has been well studied across different cancers, including pancreatic cancer. Saka et al. have extensively reviewed the mechanisms of T cell exhaustion in PDAC, with links to immunosuppressive cells, inhibitory receptors, transcriptional and epigenetic reprogramming and metabolic changes [21].

Some of the key features differentiating exhausted T cells from progenitor and memory-like T cells include niche epigenetic modification and transcriptional factor profile, upregulation of checkpoint inhibitor receptors, reduced production of anti-tumor cytokines such as IFN-γ, enhanced production of pro-tumor chemokines and an enhanced rate of apoptosis [85,86,87]. Transcriptional factors associated with an exhausted state of a T cell include nuclear factor of activated T cells (NFAT), basic leucine zipper ATF-like transcription factor (BATF), T cell factor 1 (TCF1), thymocyte selection-associated high mobility group box (TOX), and TOX2 [21]. NFAT expression is triggered by the calcium/calcineurin pathway and is highly upregulated in pancreatic cancer [88,89]. Studies have shown that NFAT binds to activator protein 1 (AP-1) at the chromatin site, which inhibits T effector function [90]. Other transcriptional factors such as BATF, a TCR signaling molecule, work alongside NFAT to achieve this exhaustion phenotype [91]. Downstream of NFAT activation, the expression of *TOX* genes is initiated. *TOX* and *TOX2* are known regulators of exhaustion as confirmed by ATAC-seq, RNA-seq, and sc-RNA-seq studies [92,93,94,95]. Transcriptional and epigenetic reprogramming that occurs by *TOX* has a ripple effect on immune checkpoint receptors, and transcriptional factors, cytokines and chemokines related to T effector cells [21]. These studies were not specific to PDAC; however, they provided insight into interconnecting mechanisms of T cell exhaustion in cancer, which is likely to be relevant to PDAC. Additionally, there are metabolic links to T cell exhaustion, such as a reduction in aerobic glycolysis. Aerobic glycolysis is crucial for the output of effector cytokine functions and is shown to be reduced in T cell exhaustion via glucose transporter type 1 (GLUT1) downregulation [96,97].

Tumor cells are one of the key contributors to T cell exhaustion in PDAC. Tumor-associated antigens are released by PDAC cells into the TME and are absorbed by antigen-presenting cells and presented to CD4+ T cells. Ongoing stimulation is associated with hyperactivation and thus contributes to exhaustion of the T cells [98]. Moreover, suppressive cytokines such as TGF-β can further promote T cell exhaustion due to metabolic stress. Ongoing exhaustion via the mechanisms described above can upregulate immune checkpoint proteins, and studies have demonstrated the expression of inhibitory exhaustion markers, including LAG3, PD-1/PD-L1, and CTLA4 [99], and galectins (i.e., gal-1 and gal-3) [21,100], on TILs, impairing their ability to produce cytokines and therefore function appropriately. Studies have also shown that the T cells in the TME are not activated to their full potential and generally possess an exhausted phenotype [101], as confirmed by human scRNA-seq and IHC studies depicting elevated levels of exhaustion markers TIGIT and CD39 on CD8+ T cells, and their corresponding receptors on cancer cells [102,103]. A recent human scRNA-seq analysis study in PDAC found the upregulation of immune regulatory genes such as *JUN*, *NFKB1* and *HSP90AA1* in CD8+ NKT-like, memory CD4+ T cells and naïve CD4+ T cell subsets [98]. These changes contribute to the reduced anti-tumor responses in the TME and thus progression of PDAC.

Of the immune subsets, TAMs are one of the key cells that contribute to T cell exhaustion in cancer. They can produce immunosuppressive factors such as TGF-β and IL-10 and prompt T cell exhaustion by upregulating PD-L1 on macrophages, which binds to PD-1 on CD8+ cytotoxic T cells [104]. Studies have shown that modulating TAMs is important in preventing the exhaustion and thus dysfunction of T cells. In a pancreatic cancer murine model, blocking macrophage recruitment via targeting CSF1/CSF1R resulted in the reprogramming of TAMs and fewer numbers present in the tumor [105]. Furthermore, the tumor significantly regressed when CSF1/CSF1R blockade was combined with PD-1/CTLA4 inhibitors and gemcitabine, highlighting the multi-target approach of CSF1/CSFR1 with other anti-tumor promoting treatments [105]. MDSCs are another key contributor to T cell exhaustion, with polymorphonuclear (PMN-MDSC) being the most common in PDAC [21]. Other myeloid subsets such as Gr-1+ CD11b+ cells were associated with the exhaustion of cytotoxic T cells via GM-CSF and CCL2 in an autochthonous mouse PDAC model [106].

T cell exhaustion is a concept explored in PDAC; however, checkpoint inhibitors remain largely unsuccessful for patients. Despite this, pre-clinical studies have shown that combination therapies such as PDA vaccine (GVAX) and CTLA-4/PD-1/PDL-1 blockade in metastatic PDAC patients improved CD8+ T cell function and IFN-γ production and overall survival [107,108]. Moreover, intratumoral in situ injection of CD40-TLR4, an important avenue for T cell immunity, has shown potential [109]. Some of the limitations of these results include limited sample numbers, studies largely done on murine models and thus not physiologically accurate, and human models showing toxic side effects such as cytokine release syndrome, liver toxicity and vascular and hematologic issues, possibly a result of human PDAC heterogeneity. Further studies in PDAC, incorporating immunosuppressive cells, inhibitory receptors, transcriptional and epigenetic reprogramming and metabolic changes that contribute to T cell exhaustion, are necessary to increase the success of immunotherapy in patients. Moreover, PDAC studies investigating the reprogramming of transcriptomic and epigenomic profiles between progenitor exhausted T cells moving towards complete irreversible exhaustion, also known as terminally exhausted T cells, are important to determine ways to salvage anti-tumor responses. In addition to T cells, further investigation into the exhaustion phenotype of other anti-tumor immune cell subsets is necessary, as we are now aware that exhaustion is beyond just the T cell population. This will give a detailed depiction and better understanding of the functional status of other key immune cell subsets.

### 4.2. Metabolic Pathways

In PDAC, tumor cells use various metabolic processes to enhance the immunosuppressive TME and promote tumor cell survival. This occurs via metabolic reprogramming and crosstalk between tumor cells and other cell subsets in the PDAC TME.

One of the key mechanisms is the production of lactate. Lactate can be produced by PDAC cells and is able to modify the function of suppressive cells such as TAMs and MDSCs. PDAC-lactate can upregulate TAM effector genes and thus TAM immunosuppressive function via metabolic processes involving hypoxia-inducible factor-1 alpha (HIF-1α) [110], histone acetylation [111] and histone lactylation [112,113]. Moreover, murine PDAC co-culture studies have shown that PDAC-specific lactate also promotes the immunosuppressive nature of MDSCs via GPR81, a receptor for lactate, signaling via HIF-1α, mechanistic target of rapamycin (mTOR) and STAT3 pathways; however, these studies need to be replicated, as they may not completely reflect that of human PDAC due to the highly heterogeneous nature of the disease [114].

In the TME of PDAC, T cells are functionally impaired by the lack of glutamine, tryptophan and arginine amino acids, as they are largely absorbed and utilized by suppressive subsets such as PDAC cells, TAMs and MDSCs to enhance their suppressive nature [115,116,117]. Moreover, the lack of glutamine means that Th1 and Th17 differentiation is impaired, but Tregs are not [118]. Collectively, this is associated with the impairment of TCR signaling, activation, memory formation and cytotoxic function of T cells [119]. Moreover, PDAC cells use vitamin B6 in the TME, which is required for NK cell glycogenolysis, thus leading to reduced cytotoxic function of the cell subset [120,121]. These are a few examples of how suppressive cells in the PDAC TME can consume metabolic factors for pro-tumor function, which would otherwise be needed for the function of immune cell subsets. Other metabolic mechanisms in the suppression of TILs include lactate dehydrogenase (LDH), 2-hydroxyglutarate, kynurenine, adenosine and lipid pathways [113].

PDAC-derived lactate can additionally modulate the function of CAFs. In vitro, CAFs absorb the lactate produced by tumor cells, initiating the expression of acetyl-CoA synthetase 2 in the PDAC cells, which is simultaneously converted to acetate by the CAFs [122,123]. In a human in vitro study, PDAC cells converted the acetate to cytosolic acetyl-CoA, linked to histone acetylation and enhanced tumor survival [123]. Furthermore, PDAC cells tell CAFs to donate essential lipids, modify amino acid metabolism, and produce nucleosides, all of which favor the tumor, enhancing its survival and progression, as nicely reviewed by Thakur et al. [113].

Targeting lactate metabolism in PDAC includes the use of LDH, key to lactate reprogramming, inhibitors such as GNE-140 and FX11, lactate transporters such as AZD3965 [124], and compounds such as BAY-8002 and AZ1729 [125]. These inhibitors have shown pre-clinical promise in promoting anti-tumor efficacy and thus inhibiting PDAC proliferation; however, they require further study, as they come with challenges such as drug instability, bioavailability and resistance [126]. These are some of the ways metabolism plays an important role in the PDAC TME and highlight the importance of metabolism in the network of cells in PDAC and their potential use in therapy.

### 4.3. Epigenetic Modification

Epigenetic modifications are a hallmark of cancer, including PDAC. There has been a recent interest in the role of epigenetic modifications in both the development and progression of PDAC, with links to modifications such as DNA methylation, histone modifications and chromatin remodeling [127,128].

DNA methylation is a process in which a methyl group is added to DNA with the aid of DNA methyltransferases [127]. This methylation process has been reported to regulate not only PDAC cells, but also TAMs and other immune cell subsets such as macrophages. Interestingly, PDAC subtypes can be identified based on DNA methylation status, with subtypes harboring distinct methylation profiles [129,130,131]. Some examples of DNA methylation in PDAC include tumor cells polarizing M1 macrophages to a pro-tumor M2 phenotype via DNA methylation suppressing glucose metabolism [132]. PDAC cells communicating with CAFs induces methylation of the immune checkpoint gene *SOCS1*, thus activating STAT3 signaling and tumor progression and metastasis [65].

Histones are proteins that DNA binds around to form a chromatin structure. Histones can modify aspects of the genome by carrying specific signals. Histone modifications can come in the main forms of acetylation and methylation, and other forms such as phosphorylation, ubiquitination and lactylation, all of which result in PDAC progression [128]. Although histone acetylation is the addition of acetyl groups to histones, in PDAC, histone deacetylases (HDACs), which remove acetyl groups, are associated with enhanced tumor progression [133,134,135]. The overexpression of HDACs correlates with the inhibition of tumor suppressor genes associated with PDAC progression. Histone methylation is the addition or removal of methyl groups to histone residues, linked to gene functional outcomes. Histone methylation is regulated by two key enzymes, lysine methyltransferases (KMTs) and lysine demethylases (KDMs). In PDAC, these enzymes are modified and linked to disease prognosis and patient outcome in human and mouse models [136,137,138]. An example of this is the overexpression of the KMT enhancer of zeste homolog 2 (EZH2), resulting in H3K27 methylation and linked to poor PDAC patient survival [139].

Chromatin remodeling is a process that transforms chromatin structure and the transcription machinery to regulate genes [140]. There are four different types of chromatin remodeling complexes: SWItch/sucrose non-fermentable (SWI/SNF), imitation SWI (ISWI), chromodomain helicase DNA-binding protein (CHD) and INOsitol-requiring mutant 80 (INO80) [141], with alterations mostly associated with deletion in SWI/SNF-related genes being the most prevalent of the four in PDAC [142]. In pancreatic cancer, one-third of patients present with aberrations or mutations linked to ATP-dependent chromatin remodeling complexes [143,144]. Some of the genes associated with this include *PBRM1*, *SETBP1*, *ARID1A* and *ARID2*, and they are known to be upregulated in cancers [128]; the alterations in the chromatin are not only linked to PDAC progression and metastasis of disease but also resistance to treatment [145,146,147].

Another way epigenetic modifications allow tumor cells to escape detection is via the downregulation or loss of human leukocyte antigen (HLA) molecules. There are different types of classical and non-classical HLA molecules, and their loss, such as in HLA class I (HLA-I), can cause a reduction in neoantigens presented to cytotoxic T cells by tumor cells, dampening an anti-tumor response [148]. Studies have shown that DNMT-mediated DNA methylation and histone-modifying enzymes are able to downregulate HLA-I, as also seen in PDAC [149,150,151,152]. Moreover, the level of HLA-I expression can be linked to patient outcomes, as shown by several human studies. A study published by Imai et al. found that 53% of PDAC patients expressed high levels of HLA-I compared to 47% with low levels, with those expressing high levels linked to improved OS and recurrence-free survival [153]. Another study found no link to OS; however, they analyzed their study based on reduced (24%) or complete loss of (6%) HLA-I expression [154]. It should be noted that these studies have low *n* values. Contrary to the above studies, a relatively more recent study by Hiraoka et al. found that low expression of HLA-I on PDAC cells was significantly associated with longer OS, mediated by IFN-γ, highlighting the heterogeneity of PDAC and the need to better understand the mechanisms behind HLA-I expression [155]. There are links established between the loss of HLA-I expression and the reduced responses in immunotherapies in cancer [156,157]. As immunotherapy responses are uncommon in PDAC at present [158,159,160], determining the mechanisms behind HLA-I expression and potentially targeting the molecule to re-trigger its expression on PDAC cells could be of benefit.

These studies yet again highlight the importance of the crosstalk between cancer cells and other subsets in the TME, and how cellular processes such as metabolism and epigenetics play a pivotal role in PDAC progression (refer to Figure 1).

## 5. Key Driver Mutations and Signaling Pathways on Immune Cells

**Figure 3:**
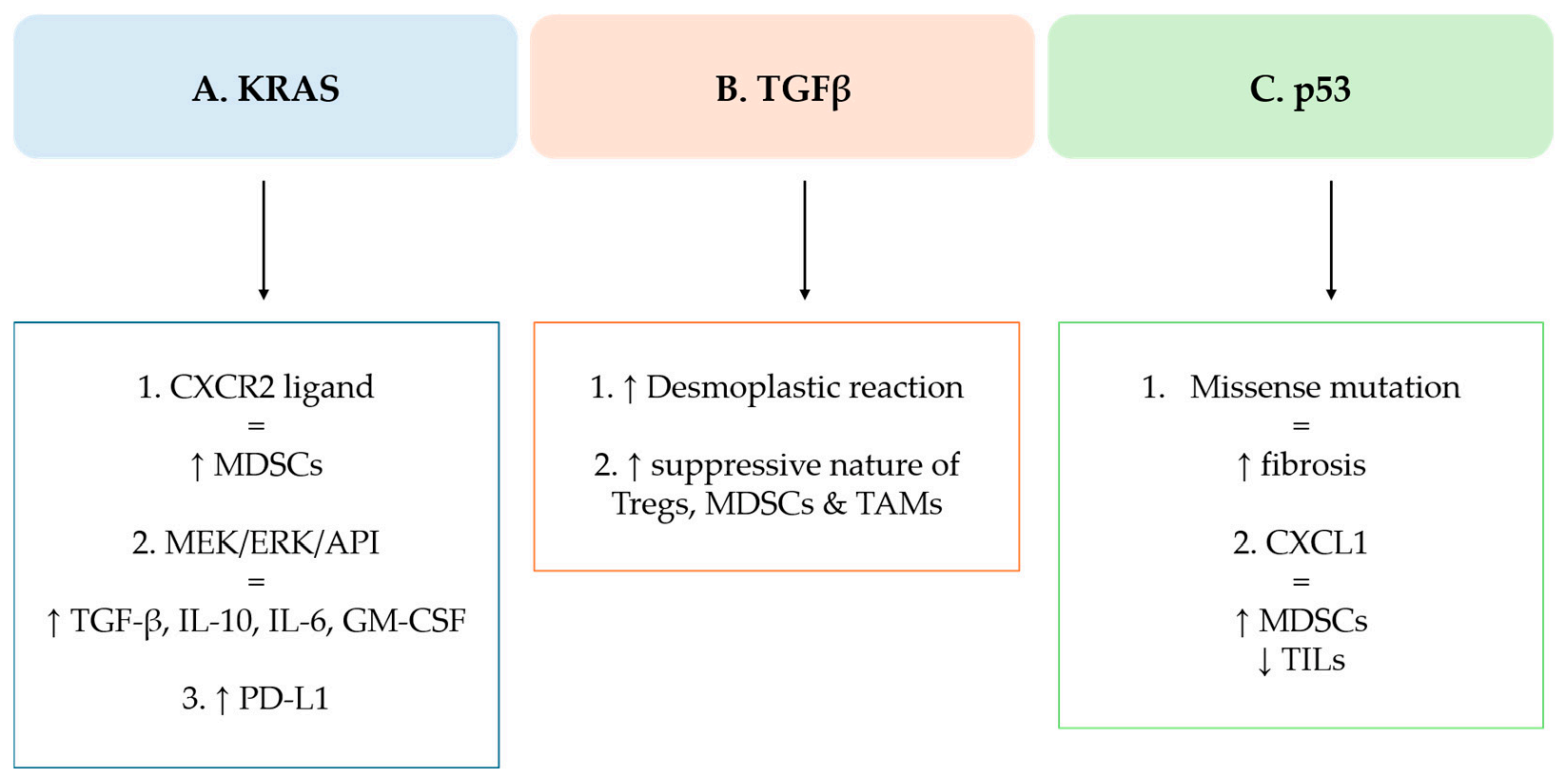
Key genetic mutations in PDAC contributing to immunosuppression. Mutations in KRAS, TGF-β and p53 signaling contribute to a pro-tumor environment, aiding in cancer progression. (**A**) *KRAS* mutations can result in CXCR2 ligand, increasing the number of MDSCs; the activation of MEK/ERK/API is associated with the upregulation of key immunosuppressive factors such as TGF-β, IL-10, IL-6 and GM-CSF, and increased PD-L1 protein expression, contributing to T cell exhaustion. (**B**) *TGF-β* mutations enhance desmoplastic reaction, which is associated with CAFs becoming more active, and the pathway can increase the suppressive nature of Tregs, MDSCs and TAMs. (**C**) *p53* mutations increase fibrosis and can create an immunosuppressive environment by mechanisms such as the chemokine CXCL1, resulting in enhanced infiltration of MDSCs and reduced TILs. ↑ represents elevation and ↓ represents reduction.

There are several different signaling pathways involved in the progression of PDAC, including KRAS, TGF-β and p53 pathways [22], working together to aid in tumor cell survival [23]. A recently published comprehensive study by Shukla et al. has identified over a thousand DEGs in PDAC tumors compared to controls [161]. This supported previous studies [162] and further confirmed the genetic signature of PDAC tumors, initiating important signaling pathways and favoring and contributing to tumorigenicity and cancer progression [161]. Figure 3 outlines the mechanisms in which mutations in the three key signaling pathways, KRAS, TGF-β and p53, contribute to an immunosuppressive TME in PDAC.

### 5.1. KRAS Signaling

Mutations in the *KRAS* gene are the most prevalent of all mutations, with cases as high as ~95% of PDAC patients, highlighting the importance of this process in the progression of cancer. The majority of the KRAS mutations occur at codon 12 (G12R, G12V, G12R, G12C, G12A and G12S) but can also be present at codon 13 (G13D, G13C, G13S and G13R) [163,164,165]. *KRAS^G12D^* constitutes the largest proportion of *KRAS* mutations (~40%) [165] (Zhang et al., 2023), and patients who carry this mutation have the least favorable outcome [166,167,168].

*KRAS* mutations not only play a pivotal role in the biology of PDAC cells, for example, by regulating key signaling pathways and cellular metabolic reprogramming [165], but they also contribute to immune escape and an immunosuppressive TME. There are several ways immune evasion is initiated, with studies demonstrating the link between *KRAS* mutations and initiation of various signaling pathways to reduce immunity in PDAC. One example is the upregulation of the chemokine CXCR2 ligand in PDAC cells from humans and mice, initiated by mutant *KRAS* and NF-κB signaling, thus recruiting immunosuppressive cells such as MDSCs into the TME and contributing to cancer progression [169]. Other ways mutant *KRAS* in PDAC cells result in immune evasion are via the production of suppressive cytokines TGF-β, IL-10, IL-6 and GM-CSF [18,170] by MEK/ERK/API pathway, which can contribute to immunosuppression in many ways, recruiting and upregulating the activity of suppressive immune cells in the TME (i.e., TAMs, Tregs, Th2) and reducing anti-tumor T cell subsets (i.e., CD8+ and CD4+ T cells) linked to factors such as the upregulation of the enzyme lipase H (LIPH) [171,172]. Moreover, *KRAS* mutations in human PDAC cells can enhance the expression of PD-L1 molecules, further promote immune escape via activating the ARF6-AMAP1 pathway in vitro [173], and reprogram CAFs to secrete cytokines that promote pro-tumor responses via the upregulation of cells like MDSCs [174]. Studies utilizing inhibitors to target *RAS G12* mutations have shown promise in PDAC. Early studies have utilized genetic models and small-molecule KRAS inhibitors in PDAC and have reported the important role of *KRAS* mutations in contributing to anti-tumor responses. Transgenic mice were used to induce and de-induce mutant *KRAS* with doxycycline [175,176]. Pancreatic tumors that formed following mutant *KRAS* shrunk when *KRAS* was de-induced, implying that PDAC tumors likely need a signal from the oncoprotein to survive [175,176]. Small-molecule inhibitor studies have found a level of reconfiguration in PDAC tumors, reducing the abundance of immunosuppressive myeloid cells and increasing the abundance and function of cytotoxic T cells [177,178]. This is of particular importance as the infiltration of T cells in the tumor is indicative of immunotherapy response, also in the context of combination use with RAS inhibitors. *KRAS* mutation mouse models using RAS inhibitor RMC-7977 or the immunotherapeutic agent CD40 agonist in combination with immune checkpoint blockade antibodies (anti-CTLA4/anti-PD-1) had enhanced effectiveness when PDAC tumors were ‘hot’ due to increased abundance of T cells, compared to ‘cold’ with fewer T cells [179]. It should be noted that these studies are pre-clinical and require further study to move into clinical trials. Recently, a phase 3 open-label, randomized clinical trial treating metastatic PDAC with Daraxonrasib, a RAS(ON) multi-selective, tri-complex inhibitor, reported that the median OS was 13.2 months, compared to 6.7 months for the chemotherapy group [19]. These results are promising; however, they are limited to the metastatic group of PDAC patients.

This highly immunosuppressive environment created by *KRAS* mutations is linked to poor prognosis for PDAC patients and justifies the need to study *KRAS* mutation as a target for PDAC in greater detail.

### 5.2. TGF-β Pathway

In normal pancreatic tissue, TGF-β signaling is responsible for maintaining homeostasis [180]. It is when the pancreatic cell becomes malignant, and in the context of PDAC, *TGF-β* and *SMAD4* become mutated, that the transformation in the pathway from a tumor suppressor to a tumor promoter occurs, with this transformation being prevalent in advanced stages of disease and not early stages (I and II) [181,182]. SMAD4 works to control TGF-β, but with its deletion or inactivation depicted in ~60% of PDAC patients [183], TGF-β can create an environment where tumor cells thrive. TGF-β/SMAD4 signaling is an example of the canonical TGF-β signaling pathway, and the non-canonical pathway, which is SMAD-independent, can also activate a series of cancer progression pathways such as PI3K/AKT and ERK/MAPK, which are known to contribute to cancer progression and EMT in cancers including PDAC [184,185,186].

In PDAC, TGF-β contributes to the desmoplastic reaction, CAF activation and EMT, playing an important role in the crosstalk between tumor cells and fibroblasts, further contributing to the immune milieu of PDAC [185,187,188,189]. This milieu is highly immunosuppressive, with TGF-β being used to dampen anti-tumor responses via the inhibition of T cells and other anti-tumor subsets [24]. Moreover, it promotes the suppressive nature of subsets like Tregs, MDSCs, TAMs and macrophages [190], and can release more TGF-β in a positive feedback loop [186]; elevated levels of TGF-β in PDAC are linked to overall immune evasion [191]. Trebska-McGown et al. found that in murine studies, TGF-β signaling can upregulate checkpoint proteins on suppressive cells, with an increase in PD-L1+ TAMs observed in PDAC [190], something that needs to be confirmed in human PDAC, as TGF signaling is a hallmark of various PDAC pathobiology processes [190].

Targeting TGF-β signaling for the treatment of PDAC is an avenue that has been explored for several years. These studies generally incorporated TGF-β pathway inhibitors in combination with chemotherapy, radiotherapy or immunotherapy to maximize the response using a multipronged approach. One study used Trabedersen, which targets the TGFβ2 transcript, in metastatic PDAC patients and reported a significant increase in OS when administered before chemotherapy and not after [192]. Galunisertib (TGFβR1 inhibitor), SHR-17011 (targeting PD-L1 and TGFβR2), M7824 (targeting PD-L1 and TGFβR2), and Vactosertib (TGF-β/SMAD inhibitor) have been used in combination with chemotherapies or immunotherapies across several PDAC studies, including clinical trial studies, as reviewed by Principe et al. [182]. Some of the clinical trial studies using SHR-17011, M7824 and Vactosertib are ongoing and have thus far shown potential; however, drug efficacy, patient risk and toxicity are some concerns with the inhibitors. Moreover, further studies into primary PDAC, and not only metastatic PDAC, are necessary to truly determine whether this treatment approach will be successful down the track.

### 5.3. p53 Pathway

Mutations in the *Tp53* tumor suppressor gene are a common trait of PDAC, with ~70% of patients harboring the mutation, with links to cancer progression [46]. In mouse studies, Siolas et al. demonstrated a mechanism of immunosuppression by *p53* mutation via the accumulation of neutrophils and PM-MDSCs when PDAC cells carry the *p53* mutation, rendering immunotherapy ineffective [193]. A comparative TCGA human PDAC study found that *p53* missense mutations were associated with poorer outcomes due to increased fibrosis and reduced TILs, when compared to *p53* wildtype and *p53* null [194]. Several gain-of-function (GOF) mutation studies in PDAC have reported links to pancreatic tumor cell migration and invasion in murine and human models, indicating that these alterations are independent of the heterogeneity observed in the disease [195,196]. These GOF mutations resulted in a 27% increase in mortality compared to non-GOF, in metastatic and locally advanced PDAC [197]. In contrast to this, another study on resected PDAC reported that tumors with GOF mutations had reduced lymphovascular invasion, and non-GOF mutations had reduced OS and disease-free survival as compared to wild type and GOF [198]. Discrepancies in locally advanced compared to resected PDAC and experimental design could account for these differences.

To establish a better understanding of the immunosuppression associated with *p53* mutations in PDAC, a new study employed a mouse model focusing on the *p53^R172H^* missense mutation. This study found a dampened anti-tumor immune response in tumors with *Trp53^R172H/−^*, with fewer T cells and greater MDSC infiltration, resulting in reduced effectiveness of immune checkpoint inhibitors [199]. The chemokine gene *CXCL1* was found to be a key contributor to the immunosuppression observed in this model. Specifically, *p53^R172H^* binds and interacts with *CXCL1*, upregulating its expression in network with NF-κB, triggering this response [199].

Mutations in these pathways are yet another way tumor cells transform the PDAC TME to be highly immunosuppressive (refer to Figure 1). As these pathway mutations can dampen anti-tumor immune responses, therapeutic studies incorporating these mutations along with immunotherapy would be of interest.

## 6. Conclusions

The immune ‘cold’ TME of cancer is a key mechanism utilized by the tumor cells to promote their aggressive nature and therefore progression. This feature is also apparent for PDAC, with immune dysregulation being a large concern. Immune dysregulation is caused by immunosuppressive cells that switch anti-tumor responses off, as well as a reactive stroma associated with CAFs and their communication with tumor cells and immune cells. Moreover, therapy resistance in PDAC, contributed to by cancer-related mechanisms such as T cell exhaustion, metabolic signaling, epigenetic modifications and genetic mutations, further contributes to immune dysregulation and works together to promote PDAC progression. Despite all the research going into PDAC, treatment remains largely unchanged and patient outcomes remain poor. Therapeutically reducing the immunosuppressive environment of PDAC from a ‘cold’ tumor to ‘hot’ remains the method of action. Further to this point, T cell exhaustion is a well-described phenomenon in PDAC; however, more research is necessary to determine the various processes leading to immune cell exhaustion in other key subsets such as NK cells. This aims to give a broader picture of the immune dysregulation in PDAC. Due to the highly multifactorial nature of PDAC and multiple inter-connecting biological pathways and mechanisms involved, targeting these pathways simultaneously may result in greater treatment efficacy. Biological systems are known to have compensatory actions which can reduce the efficacy of immunotherapy in PDAC; therefore, this multi-pronged approach may result in greater therapeutic success.

This review summarizes the key inter-connecting mechanisms behind immune dysregulation in PDAC, highlighting the need for additional research into promoting the anti-tumor immune responses in these patients. Further elucidating the specific mechanisms of this feature may lead to the discovery of new treatment options for improved outcomes for PDAC patients.

## Data Availability

No new data were created or analyzed in this study. Data sharing is not applicable to this article.

## Abbreviations

The following abbreviations are used in this manuscript:

AE	Adaptive-enriched
AIM	Activation-induced marker
AP1	Activator protein 1
apCAF	Antigen-presenting CAF
BATF	Basic leucine zipper ATF-like transcription factor
Breg	B regulatory cell
CAF	Cancer-associated fibroblast
CHD	Chromodomain helicase DNA-binding protein
DEG	Differentially expressed gene
ECM	Extracellular matrix
EMT	epithelial-to-mesenchymal transition
EZH2	Enhancer of zeste homolog 2
FAP	Fibroblast activation protein
FN1	Fibronectin 1
GLUT1	Glucose transporter 1
GM-CSF	Granulocyte-macrophage colony-stimulating factor
GOF	Gain of function
HDAC	Histone deacetylases
HIF-1α	Hypoxia-inducible factor-1 alpha
HLA-I	Human leukocyte antigen class I
iCAF	Inflammatory CAF
INO80	INOsitol-requiring mutant 80
ISWI	Imitation SWI
ITG	Integrin
KDM	Lysine demethylases
KMT	Lysine methyltransferase
KRAS	Kirsten rat sarcoma viral oncogene homolog
LDH	Lactate dehydrogenase
LIPH	Lipase H
MDSC	Myeloid-derived suppressor cell
ME	Myeloid-enriched
mTOR	Mechanistic target of rapamycin
myCAF	Myofibroblastic CAF
NET	Pancreatic neuroendocrine tumor
NFAT	Nuclear factor of activated T cell
NK cell	Natural killer cell
OS	Overall survival
PDAC	Pancreatic ductal adenocarcinoma
PDGF	Platelet-derived growth factor
PMN-MDSC	Polymorphonuclear MDSC
POSTN	Periostin
SHH	Sonic hedgehog
SPARC	Secreted protein acidic and rich in cysteine
SWI/SNF	SWItch/sucrose non-fermentable
TAM	Tumor-associated macrophage
TCF1	T cell factor 1
TCR	T cell receptor
TGF-β	Transforming growth factor-beta
THSB1	Thrombospondin 1
TIL	Tumor-infiltrating T cell
TME	Tumor microenvironment
TNC	Tenascin
TOX	Thymocyte-associated high mobility group box
Treg	T regulatory cell
TRF	Tissue-resident fibroblast
α-SMA	Alpha-smooth muscle actin
